# Effectiveness of 0.1% mometasone furoate under hydrocolloid dressing versus 0.1% mometasone furoate in patients with lichen simplex chronicus

**DOI:** 10.1002/ski2.228

**Published:** 2023-03-21

**Authors:** Hiu Lai Lo, Fong Cheng Ip

**Affiliations:** ^1^ Department of Health Social Hygiene Service Hong Kong China

## Abstract

There was a lack of high‐quality, evidence‐based treatment for lichen simplex chronicus (LSC). Topical steroid under hydrocolloid dressing treatment was investigated mostly in observational studies without investigation of the cost‐effectiveness and the methodology of application also varied without standardisation. To investigate the cost‐effectiveness of topical steroid under hydrocolloid dressing in the treatment of moderate to severe lichen simplex chronicus (LSC). The study aimed to provide a clear methodology that was replicable. A single‐blinded randomized controlled trial was carried out to compare the efficacy of 0.1% mometasone furoate cream with or without hydrocolloid dressing in patients suffering from moderate to severe LSC. Physician Global Assessment (PGA) score, Eczema Area and Severity Index (EASI) individual components score were assessed by a Dermatologist through clinical photos at week 0, 2, and 4. Pruritis Numerical Rating Scale (PNRS) was rated. Forty adult patients were recruited. The group with hydrocolloid dressing showed superior treatment efficacy. 20 out of 20 patients benefited from the hydrocolloid dressing with topical steroid while only 6 out of 20 patients benefited from topical steroid alone at week 2 regarding PGA. Similar result was obtained at week 4. Extra HK$ 132 was needed for each patient in hydrocolloid with topical steroid group. The number needed to treat (NNT) was 1.43 (95% CI: 1.42–1.44) at week 2 and 1.42 (95% CI: 1.41–1.44) at week 4 regarding PGA score improvement of ≥2. NNT analysis supported the cost‐effectiveness of adjunctive hydrocolloid dressing usage as the first‐line treatment in patients with moderate to severe LSC. This study added evidence to LSC treatment with a detailed and reproducible methodology.

## Graphical abstract

This study proved the cost effectiveness of hydrocolloid dressing usage in patient with moderate to severe lichen simplex chronicus (LSC). 0.1% mometasone furoate under hydrocolloid dressing should be considered as the first line treatment for patient with moderate to severe LSC. Moreover, behavioural treatment through habit reversal deserves further investigation on its role in rebound prevention.

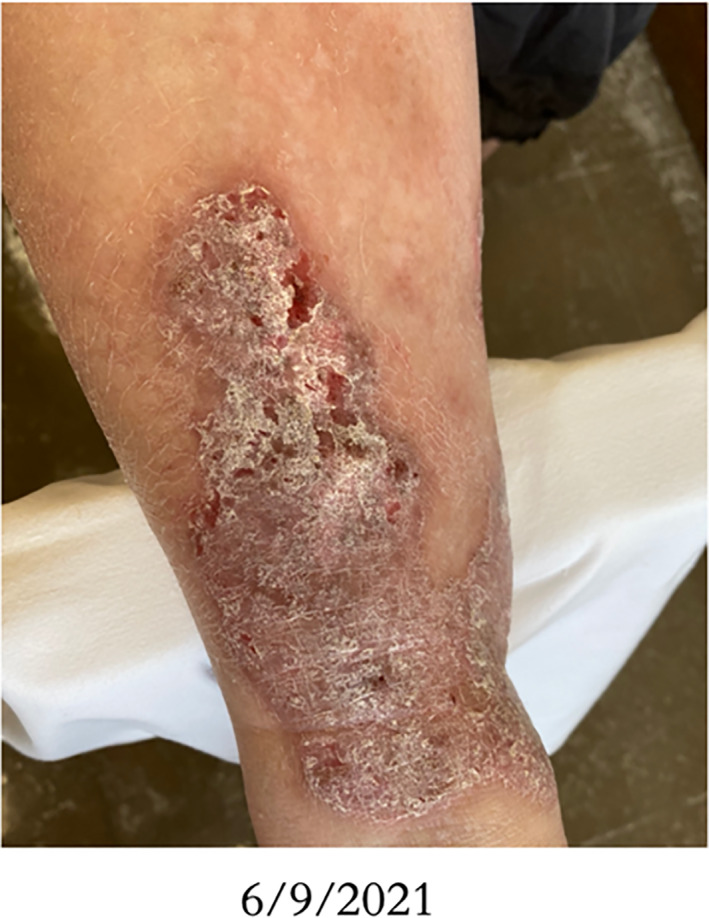

1

**What is already known about this topic?**

- Topical steroid under hydrocolloid dressing treatment was well known to be effective for the treatment of lichen simplex chronicus.

**What does this study add?**

- This study investigated the cost‐effectiveness of hydrocolloid dressing in the treatment of lichen simplex chronicus with clear methodology. It also shed some light on behavioural treatment through habit reversal.

## 2 INTRODUCTION

Lichen simplex chronicus (LSC) was a common dermatosis presented with pruritic and lichenified plaque resulting from repetitive scratching or rubbing. Neck, ankle, shin, wrist, and anogenital area were frequently involved sites.^1^, ^2^ Pruritis was found more frequently in darker skin types which might be attributed to the lower ceramide content, lower pH value, greater keratinocytes cohesion, and increased mast cells.^3^, ^4^ Psychological conditions such as depression, anxiety and obsessive‐compulsive disorder were also suggested to be related to symptoms of pruritus.^1^, ^3^, ^5^

Topical corticosteroid remained the first‐line treatment of choice for LSC.^2^, ^6^ Nevertheless, LSC could be refractory to topical steroid treatment alone. Topical steroid under hydrocolloid dressings might be the resolution. Hydrocolloid dressings composed of two layers which were the inner colloidal and outer waterproof layer. Gel‐forming agents such as cross‐linked matrix gelatin, pectin, carboxymethyl‐cellulose with hydrophilic particles, and other materials such as adhesives and elastomers were utilised. A carrier such as a sheet of polyurethane film was then added to form an absorbent, self‐adhesive, and waterproof dressing.^7^, ^8^, ^9^ Once in contact with wound exudate, the hydrocolloid absorbed and retained the exudate to form a gel. A moist environment was hence created to protect the granulation tissue formation.^9^, ^10^ However, they should be used with caution in extremities of patients with diabetes or peripheral vascular disease, as wounds in these patients were more prone to anaerobic infections.^11^

Absorption of topical steroids could be increased by several folds in the hydrated stratum corneum while topical steroids under occlusion could increase absorption from several to 10 folds.^12^, ^13^ The main possible risks of topical steroid and hydrocolloid dressing complications included allergy, contact dermatitis, hypopigmentation, hyperpigmentation, infection, hypertrichosis, acneiform eruption, skin atrophy striae, rarely hyperglycaemia, blurred vision, and adrenocortical axis suppression. Under proper clinical guidance and a short duration application, most of the possible side effects mentioned above were unlikely.

The latest systematic review of treatments for lichen simplex chronicus pointed out that there was a lack of high‐quality, evidence‐based treatments for lichen simplex chronicus.^1^ In the latest review, one RCT and one observational study examined the use of occlusion as adjunctive therapy with topical steroids showed conflicting outcomes.^1^, ^14^, ^15^ In the RCT, treatment with 0.3% diflucortolone valerate twice daily with and without plastic occlusion was investigated. The occlusive dressing group did not demonstrate therapeutic superiority.^14^ Nevertheless, in the observational study, clobetasol propionate and hydrocolloid occlusive dressing once a week led to the complete remission of disease in 89% of patients in 2 weeks.^15^ Flurandrenolone tape used in the treatment of lichen simplex chronicus was well‐known. However, duration and application method were not well documented, and the quality of the study was difficult to assess.^2^, ^16^

## 3 METHODS

**FIGURE 1:**
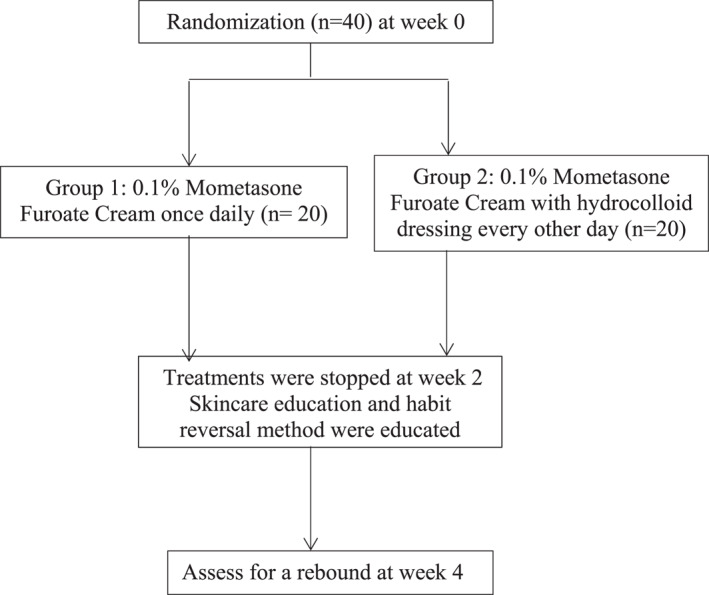
Study design flowchart.

We conducted a single‐blinded randomized controlled study on patients attending the dermatology clinics of the Social Hygiene Service under the Department of Health in Hong Kong. This study aimed to investigate the cost‐effectiveness of 0.1% mometasone furoate (Elomet^TM^ Cream) under hydrocolloid dressing (Nexcare^TM^) versus 0.1% mometasone furoate alone in patients with moderate to severe lichen simplex chronicus. It also shed some light on habit reversal methods regarding short term rebound. Randomisation was performed with shuffled sealed envelopes which contained the treatment allocation (Figure 1).

Patients aged 16–64 years would be recruited into the study if they had at least one site of lichen simplex chronicus with size ≥4 × 4 cm^2^, Physician Global Assessment (PGA) score, and Eczema Area and Severity Index (EASI) individual components score (1. Erythema 2. Papulation/oedema 3. Excoriation 4. Lichenification) equal to or more than moderate severity. Infected lichen simplex chronicus, allergy to 0.1% mometasone furoate cream or hydrocolloid dressing, pregnancy, psychiatric illness, lesions in the genital and facial area were excluded. Antihistamines and antipsychotics would not be administered during the whole study period.

One pack of Nexcare^TM^ Hydrocolloid Dressing (2 pieces 60 × 100 mm) cost HK$33. Each patient was provided 4 packs of Nexcare^TM^ Hydrocolloid Dressing with one tube of 0.1% mometasone furoate cream (HK$25) in the topical steroid with hydrocolloid dressing group while only one tube of 0.1% mometasone furoate cream (HK$25) was provided in the topical steroid only group. Extra HK$ 132 was needed for each patient in the topical steroid with hydrocolloid dressing group.

Patients with one site of LSC had the following assessment at week 0,2 and 4.

1. Physician Global Assessment (PGA) score
2. Erythema, Papulation/oedema, Excoriation, Lichenification as those individual components in Eczema Area and Severity Index (EASI) score
3. Pruritis Numerical Rating Scale (PNRS)

Trained nursing colleagues were responsible for clinical pictures record at every visit with the camera: Canon G7X Canon Zoom Lens 4.2 × IS 8.8–36.8 mm 1:1.8–2.8. A designated dermatologist not disclosed to the group allocation assessed the clinical photos and scored PGA and EASI individual components. Photos of corresponding EASI individual components severity were provided for reference. PNRS was reported by participants at every visit.

Primary endpoint: PGA score improvement ≥2.

Secondary endpoint: EASI individual components score (1. Erythema 2. Papulation/Oedema 3. Excoriation 4. Lichenification) ≥50% improvement.

Safety endpoint: Application of the topical would be terminated immediately if any adverse event appeared, such as infection, allergic reaction, skin atrophy.

Significant Pruritis Numerical Rating Scale (PNRS) improvement was defined as ≥4 points improvement in this study. ≥2–4 points change in Peak Pruritis Numerical Rating Scale was a clinically relevant within‐person response.^17^

A power of 80% and a two‐sided significance level of 95% were assumed in determining the sample size. Due to the lack of randomized control trials regarding LSC treatment with topical steroids under hydrocolloid dressing, the sample size estimation was deduced from clinical experience and a randomized, open, prospective right‐left comparison study in 19 patients with palmoplantar and localised pustular psoriasis.^18^

Intention to treat analysis with missing values replacement, using Last observation carried forward (LOCF) method was used. A participant's missing value would be replaced with the last available measurement. An independent‐samples *t*‐test was used for continuous data if data were normally distributed. In contrast, the Mann‐Whitney *U* test was used to analyse between two groups for continuous skewed data. As appropriate, categorical data were analysed by the Pearson Chi‐square test or Fisher's Exact test. The McNemar test was used to analyse the change of a categorical variable over time within the same group. A *p*‐value of <0.05 was considered statistically significant. Statistical analysis was performed using IBM SPSS Statistics for Windows, Version 26.0 (IBM Corp., Armonk. NY, USA computer software).

This study was approved by the Research Ethics Committee of the Department of Health and the Pharmacy and Prisons Board of Hong Kong.

## 4 RESULTS

Forty‐four patients with LSC were initially recruited to participate in this single‐blinded RCT study. Four patients were excluded due to their inability to meet the severity requirement after screening by the designated dermatologist. Forty patients who fulfiled the recruitment criteria were analysed. The default rate in this study was 0% at week 2% and 7.5% at week 4. No adverse event occurred in the whole study period.

In this single‐blinded RCT, the demographics of the two groups were similar with no statistically significant difference (Table 1). Age, gender, and BMI showed no significant statistical difference between groups. The median duration of LSC was 4 years in both groups. Most of our recruited cases were primary lichen simplex chronicus. The most common site involved was ankle. Table 1 summarised the demographic and physical findings.

**TABLE 1:** Demographics and physical findings.

	0.1% mometasone furoate cream (*N* = 20)	Hydrocolloid dressing with 0.1% mometasone furoate cream (*N* = 20)	*p*‐value
	Mean (SD)/Median (IQR)/*N* (%)	Mean (SD)/Median (IQR)/*N* (%)	
Age (years)	46.7 (13.6)	49.0 (12.3)	0.586
Gender			0.507
Male	8 (40%)	6 (30%)	
Female	12 (60%)	14 (70%)	
BMI	25.1 (21.8–28.3)	25.7 (21.9–31.0)	0.588
Duration (years)	4 (1.25–8.5)	4 (2.25–5.75)	0.693
Underlying eczema	2 (10%)	7 (35%)	0.127
DM (Diabetes mellitus)	4 (20%)	4 (20%)	1
Smoker	1 (5%)	2 (10%)	1
Site of LSC			0.179
Ankle	13 (65%)	11 (55%)	
Feet	2 (10%)	2 (10%)	
Shin	2 (10%)	7 (35%)	
Thigh	1 (5%)	0 (0%)	
Non lower limb	2 (10%)	0 (0%)	
PGA at week 0			0.047
PGA 4	10 (50%)	16 (80%)	
PGA 3	10 (50%)	4 (20%)	

Abbreviations: BMI, body mass index; IQR, interquartile range; LSC, lichen simplex chronicus; N, number of patients; PGA, physician global assessment; SD, standard deviation.

Intention to treat analysis with missing last observation carried forward (LOCF) method was used. A participant's missing value would be replaced with the last available measurement (Table 2).

**TABLE 2:** ITT‐with missing values replacement, using Last observation carried forward (LOCF) or imputation method.

		0.1% mometasone furoate cream		Hydrocolloid dressing with 0.1% mometasone furoate cream		*p*‐value (treatment effect‐Week2)	*p*‐value (treatment effect‐Week4)	*p*‐value (changes over time)‐0.1% mometasone furoate	*p*‐value (changes over time)‐Hydrocolloid
		Week 2 versus baseline	Week 4 versus baseline	Week 2 versus baseline	Week 4 versus baseline				
PGA	Successful	6 (30%)	5 (25%)	20 (100%)	19 (95%)	<0.001	<0.001	1	1
PNRS	Successful	8 (40%)	10 (50%)	15 (75%)	19 (95%)	0.025	0.001	0.5	0.125
Erythema	Successful	8 (40%)	9 (45%)	19 (95%)	19 (95%)	<0.001	0.001	1	1
Papulation	Successful	7 (35%)	9 (45%)	19 (95%)	20 (100%)	<0.001	<0.001	0.625	1
Excoriation	Successful	13 (65%)	15 (75%)	20 (100%)	20 (100%)	0.008	0.047	0.625	–
Lichenification	Successful	6 (30%)	7 (35%)	17 (85%)	18 (90%)	<0.001	<0.001	1	1
EASIc	Successful	6 (30%)	8 (40%)	19 (95%)	20 (100%)	<0.001	<0.001	0.625	1

Abbreviations: EASIc, Eczema Area and Severity Index individual components score total; PGA, physician global assessment; PNRS, pruritic numeric rating scale.

The group with hydrocolloid dressing showed superior treatment efficacy in all Physician Global Assessment (PGA) score, Eczema Area and Severity Index (EASI) individual components score and Pruritis Numerical Rating Scale. 20 out of 20 patients benefited from the hydrocolloid dressing with topical steroid while only 6 out of 20 patients benefited from topical steroid alone at week 2 regarding PGA. Similar result was obtained at week 4.

Change over time between week 2 and 4 was not statistically significant within‐group (*p* > 0.05) for all the above parameters. There was no short‐term rebound. Due to the low default rate, the result of intention to treat analysis and per protocol analysis were consistent.

Hydrocolloid dressing would unavoidably incur extra costs in the management of LSC. Hence, Number Needed to Treat (NNT) was calculated to justify the usage (Table 3). Each patient required 4 packs of Nexcare^TM^ Hydrocolloid Dressing for 2 weeks. One pack of Nexcare^TM^ Hydrocolloid Dressing (2 pieces of 60 × 100 mm) cost HK$ 33 while 4 packs cost HK$ 132. One tube of 0.1% mometasone furoate cream cost HK$ 25 and every patient was given one tube in both groups. The extra cost was HK $ 132 in the hydrocolloid with topical steroid group for each patient. By treating two, one could be benefited from a PGA score improvement of ≥2 at week 2 and 4.

**TABLE 3:** NNT (Intention to treat analysis, LOCF and Per protocal analysis).

	Week 2	Week 4
NNT (Intention to treat analysis, LOCF)		
PGA	1.43 (95% CI: 1.42–1.44)	1.42 (95% CI: 1.41–1.44)
PNRS	2.86 (95% CI: 2.78–2.93)	2.22 (95% CI 2.18–2.26)
EASIc	1.54 (95% CI 1.52–1.56)	1.67 (95% CI 1.65–1.69)
Erythema	1.82 (95% CI 1.79–1.84)	2.00 (95% CI 1.97–2.03)
Papulation	1.67 (95% CI 1.65–1.69)	1.81 (95% CI 1.80–1.84)
Excoriation	2.86 (95% CI 2.80–2.91)	4.00 (95% CI 3.91–4.10)
Lichenification	1.82 (95% CI 1.79–1.85)	1.82 (95% CI 1.79–1.85)
NNT (Per protocol analysis)		
PGA	1.43 (95% CI: 1.42–1.44)	1.47 (95% CI: 1.45–1.48)
PNRS	2.86 (95% CI: 2.78–2.93)	2.39 (95% CI 2.35–2.44)
EASIc	1.54 (95% CI 1.52–1.56)	1.73 (95% CI 1.71–1.75)
Erythema	1.82 (95% CI 1.79–1.84)	2.12 (95% CI 2.09–2.16)
Papulation	1.67 (95% CI 1.65–1.69)	1.90 (95% CI 1.87–1.95)
Excoriation	2.86 (95% CI 2.80–2.91)	4.75 (95% CI 4.62–4.89)
Lichenification	1.82 (95% CI 1.79–1.85)	1.58 (95% CI 1.57–1.60)

Abbreviations: EASIc, Eczema Area and Severity Index individual components score total; LOCF, last observation carried forward; NNT, number needed to treat; PGA, physician global assessment; PNRS, pruritic numeric rating scale.

## 5 DISCUSSION

Lichen simplex chronicus affected approximately 12% of the population. It occurred primarily in adults, with women more easily affected than men.^19^ The highest prevalence was in adults aged 30–50 years.^19^, ^20^, ^21^ The demographics in our study showed a similar trend. The frequently involved sites were easily reachable for scratching, such as the neck, ankle, shin, wrist, and anogenital area.^1^, ^2^ Most of our patients recruited had LSC over distal extremities (95%), especially ankle area (60%). Anogenital involvement was related to sexual dysfunction.^19^ However, the genital site was excluded in our study due to the potential topical steroid side effect upon hydrocolloid occlusion over fragile skin site.

Psychological conditions such as depression, anxiety, and obsessive‐compulsive disorder were suggestive of being related to LSC.^1^, ^3^, ^5^ It was essential to treat the underlying psychiatric problems first. Hence, patient with psychiatric diagnoses was excluded from this study. Metabolic diseases such as uncontrolled diabetes mellitus, hypertension, obesity, and peripheral vascular disease could be potential confounding factors. These confounding factors were minimised with randomisation.

The prominent histological features of LSC included epidermal hyperplasia with mostly orthokeratosis over occasional parakeratosis, wedge shape hypergranulosis, and elongation of the rete ridges. There were also a perivascular infiltrate of lymphocytes and occasional macrophages.^21^, ^22^ The orthokeratosis, especially in those moderate to severe LSC with some neural hyperplasia would require a much higher concentration of topical steroids. This could be attained by hydrocolloid dressing occlusion with the additional benefit of rapid restoration of the skin barrier to halt the perpetuation of inflammatory cascade.^7^, ^8^, ^12^, ^13^

In multiple localised inflammatory dermatoses, apart from identifying the trigger, a rapid halting of the whole inflammatory process was the key to successful treatment. The initial stimulation of the dendritic cell as antigen‐presenting cells started the whole inflammatory cascade. Naïve T cells could differentiate into self‐renewing memory T cells. Effector memory T cells entered and patrolled peripheral tissue. They could become residential in tissue such as skin to provide local surveillance.^23^, ^24^ It was then called tissue‐resident memory T‐cells. It was expected to proliferate and perpetuate the whole inflammatory cascade with the release of inflammatory cytokines. If the inflammation could be halted earlier with less resident memory T‐cell production, the disease control and prognosis were postulated to be better. Once many central memory T‐cells proliferated and circulated in the blood, they might enter different peripheral tissues and become resident memory T‐cells in another part of the skin.^23^, ^25^ This might be the contributing or perpetuating factor for various inflammatory skin conditions.

Intralesional steroids might be a method to halt the underlying inflammatory condition and cease the itch rapidly. Triamcinolone acetonide 2.5 mg/ml administered in a dose of 7.5–20 mg intralesional every 3–4 weeks was believed to be safe.^2^ However, the intralesional injection caused significant pain, especially when multiple injections were required every few weeks. Hydrocolloid dressing potentiated the effect of topical steroids from several to 10 folds.^12^, ^13^ This might be an effective, painless treatment alternative to intralesional steroid.

Other topical treatments options such as doxepin cream, capsaicin cream, tacrolimus ointment, and pimecrolimus cream were available.^2^ However, the itch‐scratch cycle with rebound was still a non‐resolving problem. Topical calcineurin inhibitor had the advantage of not causing skin atrophy. It was a potential candidate to be used under hydrocolloid dressing occlusion if systemic absorption could be proved negligible. There was a concern regarding the possible increased risk of lymphoma. However, there had been emerging evidence of a negligible association between cancer risk and topical calcineurin inhibitors.

Treatment such as transcutaneous electrical stimulation and acupuncture/electroacupuncture had been documented. However, the professional staffing and equipment were limited. Behavioural treatment through habit reversal was easy to carry out in an outpatient setting. It caused no harm and extra cost despite its weak evidence. All patients recruited in the study were educated at week 2 to substitute a competing response for the urge to scratch.^2^ Patting, applying emollient, and even clenching of the fist were distraction methods to replace the scratching action.

A dermatology specialist was responsible for the PGA score and EASI individual components score assessment. He was blinded to the group allocation of study patients to decrease the observer bias. Two sets of clinical photos over the study site were taken by trained clinic nursing colleagues for each patient in each visit to decrease the possibility of intra‐observer bias.

**FIGURE 2:**
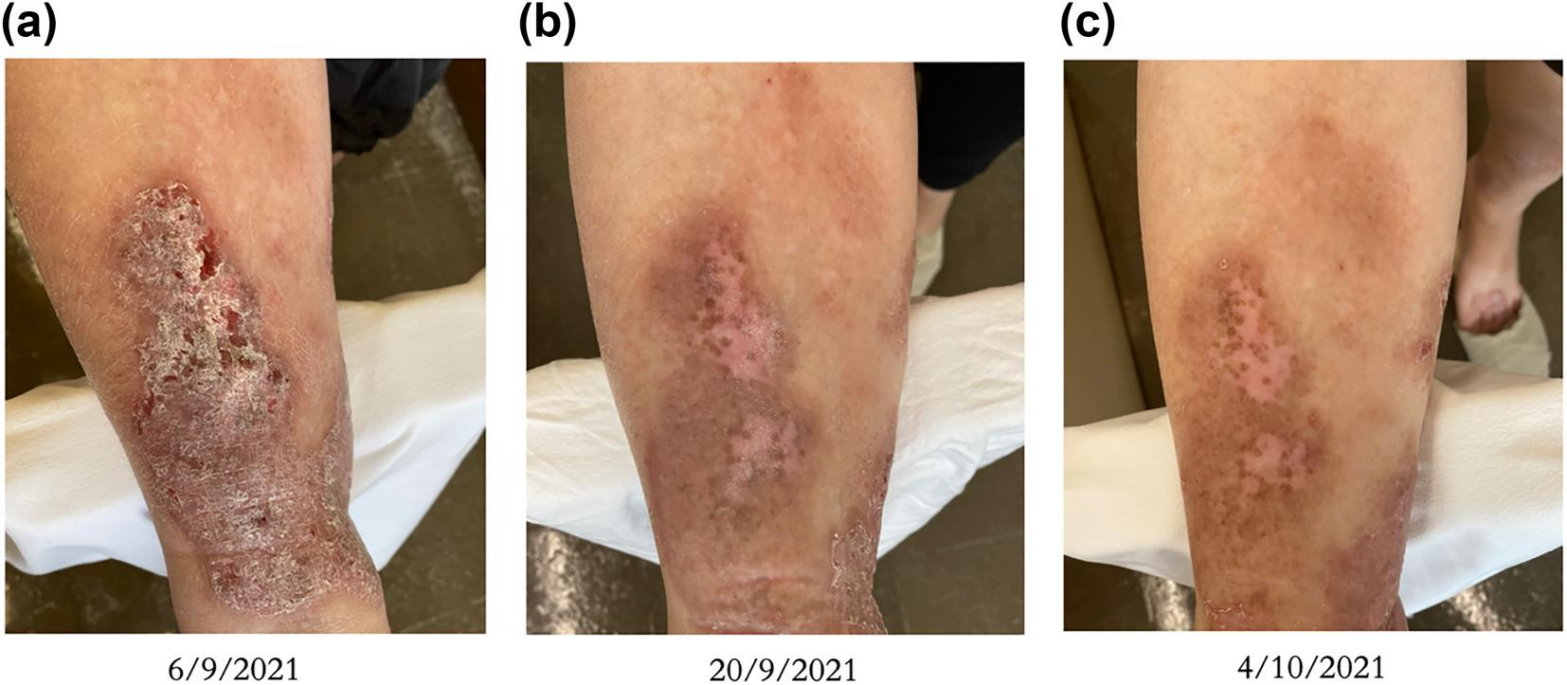
Case example of 0.1% mometasone furoate cream under hydrocolloid dressing. (a) At week 0, a 55‐year‐old female with 3 years of LSC over right shin was started on treatment with 0.1% mometasone furoate cream under hydrocolloid dressing; (b) At week 2, treatment with 0.1% mometasone furoate cream under hydrocolloid dressing was stopped. Habit reversal methods was educated and only emollient was applied for extra 2 weeks; (c) At week 4, further improvement with no rebound of skin condition was noted.

One of the concerns regarding hydrocolloid dressing usage was the cost. In this study, only extra HK$ 132 was needed per patient in topical steroid with hydrocolloid dressing group. Less than 2 patients were needed to be treated for a single patient to benefit from a PGA score improvement of ≥2 at week 2 and 4. This more efficacious treatment with hydrocolloid dressing would lead to a shorter treatment time and less visit to the clinic. The cost‐effectiveness of extra hydrocolloid dressing usage was justified (Figure 2).

Lichen simplex chronic was notorious for a rebound after stopping treatment. A potent topical steroid under occlusion was postulated to halt the inflammatory cascade effectively. The extra wound healing and barrier repair effect of hydrocolloid dressing was expected to lower the chance of a rebound. Further improvements were recorded in some cases with emollient application and distraction method education alone despite the improvements were not statistically significant. To further study the therapeutic effect, it would be valuable to add on a 3–6 months follow up to observe for any clinical rebound in both groups.

## 6 CONCLUSION

In this study of treating patients with moderate to severe lichen simplex chronicus, 0.1% mometasone furoate cream under hydrocolloid dressing showed statistically significant superiority over 0.1% mometasone furoate cream alone regarding PGA, EASI individual components score, and PNRS. NNT analysis supported the cost‐effectiveness of adjunctive hydrocolloid dressing usage. This randomized control trial added evidence to LSC treatment with a detailed and reproducible methodology.

## CONFLICT OF INTEREST STATEMENT

None to declare.

## AUTHOR CONTRIBUTIONS

**Hiu Lai Lo**: Conceptualization (Lead); Data curation (Lead); Formal analysis (Lead); Funding acquisition (Lead); Investigation (Lead); Methodology (Lead); Project administration (Lead); Resources (Lead); Software (Lead); Supervision (Lead); Validation (Lead); Visualization (Lead); Writing – original draft (Lead); Writing – review & editing (Lead). **Fong Cheng Ip**: Conceptualization (Supporting); Data curation (Supporting); Formal analysis (Supporting); Funding acquisition (Supporting); Investigation (Supporting); Methodology (Supporting); Project administration (Supporting); Resources (Supporting); Software (Supporting); Supervision (Supporting); Validation (Supporting); Visualization (Supporting); Writing – original draft (Supporting); Writing – review & editing (Supporting).

## ETHICS STATEMENT

The study was approved by Department of Health (Hong Kong) and its Ethics Committee.

## Data Availability

The data that support the findings of this study are available from Department of Health (Hong Kong). Restrictions apply to the availability of these data, which were used under license for this study. Data are available from the authors with the permission of Department of Health (Hong Kong).
